# Supplementary material for: The Association between Vaginal Dysbiosis and Reproductive Outcomes in Sub-Fertile Women Undergoing IVF-Treatment: A Systematic PRISMA Review and Meta-Analysis
Source: Pathogens. 2021 Mar 4;10(3):295. doi: 10.3390/pathogens10030295 (PMC8001118; doi:10.3390/pathogens10030295)
Supplement: Supplementary file 1 [file pathogens-10-00295-s001.pdf]

Table of contents

Supplementary Material 1. Literature search. **3**

Supplementary Material 2. New Castle – Ottawa Quality Assessment Scale - Cohort studies **4**

Supplementary Material 3. Study Characteristic (VD prevalence) **7**

Supplementary Material 4. Live birth rate (Total) **8**

Supplementary Material 4. Live birth rate (Molecular) *8*

Supplementary Material 4. Live birth rate (Microscopy) *8*

Supplementary Material 5. Early pregnancy loss (Total) **9**

Supplementary Material 5. Early pregnancy loss (Molecular) *9*

Supplementary Material 5. Early pregnancy loss (Microscopy) *9*

Supplementary Material 6. Clinical pregnancy (Total) **10**

Supplementary Material 6. Clinical pregnancy (Molecular) *10*

Supplementary Material 6. Clinical pregnancy (Microscopy) *10*

Supplementary Material 7. Biochemical pregnancy (Total) **11**

Supplementary Material 7. Biochemical pregnancy (Molecular) *11*

Supplementary Material 7. Biochemical pregnancy (Microscopy) *11*

Supplementary Material 8. Forest plot. Live birth rate (Total) **12**

Supplementary Material 8. Forest plot. Live birth rate (Molecular) *12*

Supplementary Material 8. Forest plot. Live birth rate (Microscopy) *12*

Supplementary Material 9. Forest plot. Early pregnancy loss (Total) **13**

Supplementary Material 9. Forest plot. Early pregnancy loss (Molecular) *13*

Supplementary Material 9. Forest plot. Early pregnancy loss (Microscopy) *13*

Supplementary Material 10. Forest plot. Clinical pregnancy (Total) **14**

Supplementary Material 10. Forest plot. Clinical pregnancy (Molecular) *14*

Supplementary Material 10. Forest plot. Clinical pregnancy (Microscopy) *14*

Supplementary Material 11. Forest plot. Biochemical pregnancy (Total) **15**

Supplementary Material 11. Forest plot. Biochemical pregnancy (Molecular) *15*

Supplementary Material 11. Forest plot. Biochemical pregnancy (Microscopy) *15*

Supplementary Material 12. Funnel plot. **16**

Live birth rate ... 16
Early pregnancy loss ... 17
Clinical pregnancy. ... 18
Biochemical pregnancy. ... 19
Supplementary Material 13. Moose guidelines and PRISMA Guideline. ... **20**
MOOSE guideline ... 20
PRISMA Guideline ... 21
Supplementary Material 14. GRADE. Quality of evidence ... **23**

Supplementary Material 1. Literature search.

September 27. 2020. 528 hits.

(((((((((((((IVF) OR in vitro fertilization) OR fertilization in vitro)) OR (((infertile women) OR infertility) OR female infertility))) AND (("vaginitis"[MeSH Terms] OR "vaginitis"[All Fields]) OR ("vaginal diseases"[MeSH Terms] OR ("vaginal"[All Fields] AND "diseases"[All Fields]) OR "vaginal diseases"[All Fields] OR "vaginosis"[All Fields]) OR "vaginal microbiome"[All Fields] OR "vaginal microbiota"[All Fields] OR "vaginal infection"[All Fields] OR "vaginal infections"[All Fields] OR ("vaginosis, bacterial"[MeSH Terms] OR ("vaginosis"[All Fields] AND "bacterial"[All Fields]) OR "bacterial vaginosis"[All Fields] OR ("bacterial"[All Fields] AND "vaginosis"[All Fields])) OR ("vaginosis, bacterial"[MeSH Terms] OR ("vaginosis"[All Fields] AND "bacterial"[All Fields]) OR "bacterial vaginosis"[All Fields] OR ("bacterial"[All Fields] AND "vaginitis"[All Fields]) OR "bacterial vaginitis"[All Fields])))) NOT ((((((((IVF) OR in vitro fertilization) OR fertilization in vitro)) OR (((infertile women) OR infertility) OR female infertility))) AND (("vaginitis"[MeSH Terms] OR "vaginitis"[All Fields]) OR ("vaginal diseases"[MeSH Terms] OR ("vaginal"[All Fields] AND "diseases"[All Fields]) OR "vaginal diseases"[All Fields] OR "vaginosis"[All Fields]) OR "vaginal microbiome"[All Fields] OR "vaginal microbiota"[All Fields] OR "vaginal infection"[All Fields] OR "vaginal infections"[All Fields] OR ("vaginosis, bacterial"[MeSH Terms] OR ("vaginosis"[All Fields] AND "bacterial"[All Fields]) OR "bacterial vaginosis"[All Fields] OR ("bacterial"[All Fields] AND "vaginosis"[All Fields])) OR ("vaginosis, bacterial"[MeSH Terms] OR ("vaginosis"[All Fields] AND "bacterial"[All Fields]) OR "bacterial vaginosis"[All Fields] OR ("bacterial"[All Fields] AND "vaginitis"[All Fields]) OR "bacterial vaginitis"[All Fields]))) AND Review[ptyp]))) NOT ((((((((((IVF) OR in vitro fertilization) OR fertilization in vitro)) OR (((infertile women) OR infertility) OR female infertility))) AND (("vaginitis"[MeSH Terms] OR "vaginitis"[All Fields]) OR ("vaginal diseases"[MeSH Terms] OR ("vaginal"[All Fields] AND "diseases"[All Fields]) OR "vaginal diseases"[All Fields] OR "vaginosis"[All Fields]) OR "vaginal microbiome"[All Fields] OR "vaginal microbiota"[All Fields] OR "vaginal infection"[All Fields] OR "vaginal infections"[All Fields] OR ("vaginosis, bacterial"[MeSH Terms] OR ("vaginosis"[All Fields] AND "bacterial"[All Fields]) OR "bacterial vaginosis"[All Fields] OR ("bacterial"[All Fields] AND "vaginosis"[All Fields])) OR ("vaginosis, bacterial"[MeSH Terms] OR ("vaginosis"[All Fields] AND "bacterial"[All Fields]) OR "bacterial vaginosis"[All Fields] OR ("bacterial"[All Fields] AND "vaginitis"[All Fields]) OR "bacterial vaginitis"[All Fields])))) NOT ((((((((IVF) OR in vitro fertilization) OR fertilization in vitro)) OR (((infertile women) OR infertility) OR female infertility))) AND (("vaginitis"[MeSH Terms] OR "vaginitis"[All Fields]) OR ("vaginal diseases"[MeSH Terms] OR ("vaginal"[All Fields] AND "diseases"[All Fields]) OR "vaginal diseases"[All Fields] OR "vaginosis"[All Fields]) OR "vaginal microbiome"[All Fields] OR "vaginal microbiota"[All Fields] OR "vaginal infection"[All Fields] OR "vaginal infections"[All Fields] OR ("vaginosis, bacterial"[MeSH Terms] OR ("vaginosis"[All Fields] AND "bacterial"[All Fields]) OR "bacterial vaginosis"[All Fields] OR ("bacterial"[All Fields] AND "vaginosis"[All Fields])) OR ("vaginosis, bacterial"[MeSH Terms] OR ("vaginosis"[All Fields] AND "bacterial"[All Fields]) OR "bacterial vaginosis"[All Fields] OR ("bacterial"[All Fields] AND "vaginitis"[All Fields]) OR "bacterial vaginitis"[All Fields]))) AND Review[ptyp])) AND Animals[Mesh:noexp])))) AND English[lang]

Supplementary Material 2. New Castle – Ottawa Quality Assessment Scale - Cohort studies

Study 1
Bernabeu, A. *et al.* Effect of the vaginal microbiome on the pregnancy rate in women receiving assisted reproductive treatment. *Journal of assisted reproduction and genetics* **36**, 2111-2119, doi:10.1007/s10815-019-01564-0 (2019).

A. Selection
1) Representativeness of the exposed cohort: Good. Cohort came from Spain.

Although the cause of infertility has not been described.

2) Selection of the non-exposed cohort: Good, drawn from the same population as the exposed cohort.

3) Ascertainment of exposure: Good. Vaginal sample collection well defined. Use of NGS (16s rRNA) to analyze data.

4) Demonstration that outcome of interest was not present at start of study: yes

B. Comparability
1) Comparability of cohorts on the basis of the design or analysis: good, the endometrial preparation protocol of the study helped to normalize the cohort of the study.

C. Outcome
1) Assessment of outcome: Good.

2) Was follow-up long enough for outcomes to occur: Yes.

3) Adequacy of follow up of cohorts: Yes, on both biochemical and clinical pregnancies, however data regarding LBR on two subject (18%) who were verified clinical pregnant.

Total score = 4 + 1 + 2 = 7/9

Study 2
Kyono, K., Hashimoto, T., Nagai, Y. & Sakuraba, Y. Analysis of endometrial microbiota by 16S ribosomal RNA gene sequencing among infertile patients: a single-center pilot study. *Reproductive medicine and biology* 17, 297-306, doi:10.1002/rmb2.12105 (2018).

A. Selection
1) Representativeness of the exposed cohort: Study population from Japan.

2) Selection of the non-exposed cohort: Good, drawn from the same population as the exposed cohort.

3) Ascertainment of exposure: Good. Vaginal samples collected in different menstrual cycles. Use of NGS (16s rRNA) to analyze data.

4) Demonstration that outcome of interest was not present at start of study: yes

B. Comparability

1) Comparability of cohorts on the basis of the design or analysis: Poor, the article does not compare groups in relation to vaginal flora. Comparison is age dependent; >38 and <38. Limited sample size.

C. Outcome

1) Assessment of outcome: Poor description of the outcome data.

2) Was follow-up long enough for outcomes to occur: Only limited data on outcome, biochemical pregnancy, LBR and spontaneous abortion not thoroughly reported.

3) Adequacy of follow up of cohorts: A short follow up period. During the study period 15 out of 79 IVF patients achieved pregnancy. A longer follow-up period would have given data on different outcomes.

Total score = 3 + 0 + 0 = 3/9

Study 3

Vergaro, P. *et al.* Vaginal microbiota profile at the time of embryo transfer does not affect live birth rate in IVF cycles with donated oocytes. *Reproductive biomedicine online* **38**, 883-891, doi:10.1016/j.rbmo.2018.12.019 (2019).

A. Selection

1) Representativeness of the exposed cohort: Good. Cohort came from Spain.

2) Selection of the non-exposed cohort: Good, drawn from the same population as the exposed cohort.

3) Ascertainment of exposure: Good. Vaginal sample collection well defined. Used qPCR to analyze data.

4) Demonstration that outcome of interest was not present at start of study: yes

B. Comparability

1) Comparability of cohorts on the basis of the design or analysis: good

C. Outcome

1) Assessment of outcome: Good.

2) Was follow-up long enough for outcomes to occur: Yes. All outcomes reported.

3) Adequacy of follow up of cohorts: yes.

Total score = 4 + 1 + 3 = 8/9

Study 4

Koedooder, R. *et al.* The vaginal microbiome as a predictor for outcome of in vitro fertilization with or without intracytoplasmic sperm injection: a prospective study. *Human reproduction (Oxford, England)* **34**, 1042-1054, doi:10.1093/humrep/dez065 (2019).

A. Selection

1) Representativeness of the exposed cohort: Good. Cohort from Greece. Long interval between vaginal swab and ET.

2) Selection of the non-exposed cohort: Good, drawn from the same population as the exposed cohort.

3) Ascertainment of exposure: Good. Vaginal sample collection well defined. Used IS-pro technique used.

4) Demonstration that outcome of interest was not present at start of study: yes

B. Comparability
1) Comparability of cohorts on the basis of the design or analysis: Good.

C. Outcome

1) Assessment of outcome: Fine, ultrasound proven heartbeat.

2) Was follow-up long enough for outcomes to occur: Did not report miscarriage, LBR and biochemical pregnancy.

3) Adequacy of follow up of cohorts: no.

Total score = 4 + 1 + 1 = 6/9

Study 5
Moragianni, D. *et al.* Genital tract infection and associated factors affect the reproductive outcome in fertile females and females undergoing in vitro fertilization. *Biomedical reports* **10**, 231-237, doi:10.3892/br.2019.1194 (2019).

A. Selection

1) Representativeness of the exposed cohort: Good. Cohort from Greece.

2) Selection of the non-exposed cohort: Good. Fertile woman with at least one child.

3) Ascertainment of exposure: Poor. Vaginal sample collection was not described. Used Nugent scoring system.

4) Demonstration that outcome of interest was not present at start of study: yes

B. Comparability
1) Comparability of cohorts on the basis of the design or analysis: Good.

C. Outcome

1) Assessment of outcome: Fine, ultrasound proven heartbeat and HCG concentration at week 8.

2) Was follow-up long enough for outcomes to occur: Yes.

3) Adequacy of follow up of cohorts: The follow-up period is not listed in the article, but authors were contacted and provided relevant information.

Total score = 3 + 1 + 3 = 7/9

Supplementary Material 3. Study Characteristic (VD prevalence)

Studies	No. Participants (microbiom)	No. Participants (Microscopy)	Total no. participants	VD (Microbiom)	VD (Microscopy)	BV (total)
Haahr et al.	130	130	130	36	27	36
Mangot-Bertrand et al.	307	307	307	29	17	29
Selim et al.	-	71	71	-	26	26
Eckert et al.	-	91	91	-	10	10
Liversedge et al.	-	301	301	-	77	77
Gaudoin et al.	-	246	246	-	40	40
Boomsma et al.	-	197	197	-	17	17
Eldivan et al.	-	45	45	-	17	17
Moini et al.	-	399	399	-	29	29
Moore et al.	-	91	91	-	12	12
Ralph et al.	-	771	771	-	190	190
Spandorfer et al.	-	331	331	-	14	14
Moragianni et al.	-	111	111	-	41	41
Vergaro et al.	150	-	150	35	-	35
Koedooder et al	192	-	192	34	-	34
Kyono et al.	79	-	79	35	-	35
Bernabeu et al.	31	-	31	2	-	2
**Total**	**889**	**3091**	**3543**	**171**	**517**	**644**

Supplementary Material 4. Live birth rate (Total)

Studies	Analysis method	Chance of getting pregnant		No. Participants	
		AVM	NVM	AVM	NVM
Haahr et al.	qPCR	2 (11)	25 (45)	18	57
Mangot-Bertrand et al.	qPCR - Nugent score	7 (28)	67 (24)	28	262
Liversedge et al.	Nugent score	20 (27)	50 (24)	75	212
Moini et al.	Nugent score	11 (44)	102 (33)	25	311
Moore et al.	Nugent score	2(10)	25(31)	10	81
Spandorfer et al.	Nugent score	8(58)	123(39)	14	317
Vergaro et al.	qPCR	11(31)	45 (39)	35	114
Bernabeu et al.	16S (microbiom)	1(50)	8(30)	2	27
Moragianni et al.	Nugent score	10	22	41	70
	**Total**	**72**	**467**	**248**	**1451**

Supplementary Material 4. Live birth rate (Molecular)

Studies	Analysis method	Chance of getting pregnant		No. Participants	
		AVM	NVM	AVM	NVM
Haahr et al.	qPCR	2	25	18	57
Mangot-Bertrand et al.	qPCR - Nugent score	7	67	28	262
Vergaro et al.	qPCR	11	45	35	114
Bernabeu et al.	16S (microbiom)	1	8	2	27
	**Total**	**21**	**145**	**83**	**460**

Supplementary Material 4. Live birth rate (Microscopy)

Studies	Analysis method	Chance of getting pregnant		No. Participants	
		AVM	NVM	AVM	NVM
Haahr et al.	Nugent Score	1	26	11	64
Liversedge et al.	Nugent score	20	50	75	212
Moini et al.	Nugent score	11	102	25	311
Moore et al.	Nugent score	2	25	10	81
Spandorfer et al.	Nugent score	8	123	14	317
Moragianni et al.	Nugent score	10	22	41	70
	**Total**	**52**	**348**	**176**	**1055**

Table legend: AVM=Abnormal vaginal microbiota, NVM=Normal vaginal microbiota

Supplementary Material 5. Early pregnancy loss (Total)

		Biochemical - Clinical pregnancy		No. Participants	
**Studies**	**Analysis method**	**AVM**	**NVM**	**AVM**	**NVM**
Haahr et al.	qPCR	4(67)	5 (16)	6	32
Mangot-Bertrand et al.	qPCR - Nugent score	1 (13)	25 (25)	8	101
Selim et al.	Nugent score	2 (35)	2 (47)	9	21
Eckert et al.	Nugent score	1 (33)	13 (34)	3	38
Liversedge et al.	Nugent score	4 (17)	9 (14)	24	64
Gaudoin et al.	Nugent score	3 (30)	19 (26)	10	72
Boomsma et al.	Nugent score	2 (25)	18 (31)	8	59
Eldivan et al.	Nugent score	0(0)	0(0)	4	12
Moini et al.	Nugent score	4 (27)	19 (15)	15	123
Ralph et al.	Nugent score	22 (36)	34 (19)	61	176
Spandorfer et al.	Nugent score	2(14)	28 (9)	14	317
Moragianni et al.	Nugent score	0	0	41	70
Vergaro et al.	qPCR	6(35)	11 (17)	17	64
Bernabeu et al.	16S (microbiom)	0(0)	1(7)	2	15
**Total**		**51**	**184**	**222**	**1164**

Supplementary Material 5. Early pregnancy loss (Molecular)

		Biochemical - Clinical pregnancy		No. Participants	
**Studies**	**Analysis method**	**AVM**	**NVM**	**AVM**	**NVM**
Haahr et al.	qPCR	4	5	6	32
Mangot-Bertrand et al.	qPCR - Nugent score	1	25	8	101
Vergaro et al.	qPCR	6	11	17	64
Bernabeu et al.	16S (microbiom)	0	1	2	15
**Total**		**11**	**42**	**33**	**212**

Supplementary Material 5. Early pregnancy loss (Microscopy)

		Biochemical - Clinical pregnancy		No. Participants	
**Studies**	**Analysis method**	**AVM**	**NVM**	**AVM**	**NVM**
Haahr et al.	Nugent score	1	6	2	36
Selim et al.	Nugent score	2	2	9	21
Eckert et al.	Nugent score	1	13	3	38
Liversedge et al.	Nugent score	4	9	24	64
Gaudoin et al.	Nugent score	3	19	10	72
Boomsma et al.	Nugent score	2	18	8	59
Eldivan et al.	Nugent score	0	0	4	12
Moini et al.	Nugent score	4	19	15	123
Ralph et al.	Nugent score	22	34	61	176
Spandorfer et al.	Nugent score	2	28	14	317
Moragianni et al.	Nugent score	0	0	41	70
**Total**		**41**	**148**	**191**	**988**

Table legend: AVM=Abnormal vaginal microbiota, NVM=Normal vaginal microbiota

Supplementary Material 6. Clinical pregnancy (Total)

		Chance of getting pregnant		No. Participants	
**Studies**	**Analysis method**	**AVM**	**NVM**	**AVM**	**NVM**
Haahr et al.	qPCR	2 (9)	27 (44)	22	62
Mangot-Bertrand et al.	qPCR - Nugent score	8 (29)	92 (35)	28	262
Selim et al.	Nugent score	7 (27)	19 (42)	26	45
Eckert et al.	Nugent score	2 (20)	25 (31)	10	81
Liversedge et al.	Nugent score	24 (32)	64 (30)	75	212
Gaudoin et al.	Nugent score	7 (18)	53 (26)	40	206
Boomsma et al.	Nugent score	6 (35)	41 (23)	17	180
Eldivan et al.	Nugent score	4(24)	12 (43)	17	28
Moini et al.	Nugent score	12 (48)	108 (35)	25	311
Moore et al.	Nugent score	2(10)	25(31)	10	81
Ralph et al.	Nugent score	41(22)	122 (25)	190	493
Spandorfer et al.	Nugent score	8(58)	147(46)	14	317
Moragianni et al.	Nugent score	10 (24)	22 (31)	41	70
Vergaro et al.	qPCR	11(31)	53 (46)	35	115
Koedooder et al	IS-pro (microbiom)	2(6)	65(41)	34	158
Kyono et al.	16S (microbiom)	5(14)	9(32)	35	44
Bernabeu et al.	16S (microbiom)	1 (50)	15 (52)	2	29
**Total**		**152**	**899**	**621**	**2694**

Supplementary Material 6. Clinical pregnancy (Molecular)

		Chance of getting pregnant		No. Participants	
**Studies**	**Analysis method**	**AVM**	**NVM**	**AVM**	**NVM**
Haahr et al.	qPCR	2	27	22	62
Mangot-Bertrand et al.	qPCR - Nugent score	8	92	28	262
Vergaro et al.	qPCR	11	53	35	115
Koedooder et al	IS-pro (microbiom)	2	65	34	158
Kyono et al.	16S (microbiom)	5	9	35	44
Bernabeu et al.	16S (microbiom)	1	15	2	29
**Total**		**29**	**261**	**156**	**670**

Supplementary Material 6. Clinical pregnancy (Microscopy)

		Chance of getting pregnant		No. Participants	
**Studies**	**Analysis method**	**AVM**	**NVM**	**AVM**	**NVM**
Haahr et al.	Nugent score	1	28	12	72
Selim et al.	Nugent score	7	19	26	45
Eckert et al.	Nugent score	2	25	10	81
Liversedge et al.	Nugent score	24	64	75	212
Gaudoin et al.	Nugent score	7	53	40	206
Boomsma et al.	Nugent score	6	41	17	180
Eldivan et al.	Nugent score	4	12	17	28
Moini et al.	Nugent score	12	108	25	311
Moore et al.	Nugent score	2	25	10	81
Ralph et al.	Nugent score	41	122	190	493
Spandorfer et al.	Nugent score	8	147	14	317
Moragianni et al.	Nugent score	10	22	41	70
**Total**		**124**	**666**	**477**	**2096**

Table legend: AVM=Abnormal vaginal microbiota, NVM=Normal vaginal microbiota

Supplementary Material 7. Biochemical pregnancy (Total)

		Chance of getting pregnant		No. Participants	
**Studies**	**Analysis method**	**AVM**	**NVM**	**AVM**	**NVM**
Haahr et al.	qPCR	6(27)	32(52)	22	62
Mangot-Bertrand et al.	qPCR - Nugent score	8 (29)	101 (39)	28	262
Selim et al.	Nugent score	9 (35)	21 (47)	26	45
Eckert et al.	Nugent score	3 (30)	38 (47)	10	81
Gaudoin et al.	Nugent score	10 (25)	72 (35)	40	206
Boomsma et al.	Nugent score	8 (47)	59 (33)	17	180
Eldivan et al.	Nugent score	4(24)	12 (43)	17	28
Moini et al.	Nugent score	15 (60)	123 (40)	25	311
Moore et al.	Nugent score	2(10)	25(31)	10	81
Ralph et al.	Nugent score	61(32)	176 (30)	190	581
Spandorfer et al.	Nugent score	10(71)	175(55)	14	317
Moragianni et al.	Nugent score	10 (24)	22 (31)	41	70
Vergaro et al.	qPCR	17(49)	64 (56)	35	115
Bernabeu et al.	16S (microbiom)	1(50)	16(55)	2	29
**Total**		**164**	**936**	**477**	**2368**

Supplementary Material 7. Biochemical pregnancy (Molecular)

		Chance of getting pregnant		No. Participants	
**Studies**	**Analysis method**	**AVM**	**NVM**	**AVM**	**NVM**
Haahr et al.	qPCR	6	32	22	62
Mangot-Bertrand et al.	qPCR - Nugent score	8	101	28	262
Vergaro et al.	qPCR	17	64	35	115
Bernabeu et al.	16S (microbiom)	1	16	2	29
**Total**		**32**	**213**	**87**	**468**

Supplementary Material 7. Biochemical pregnancy (Microscopy)

		Chance of getting pregnant		No. Participants	
**Studies**	**Analysis method**	**AVM**	**NVM**	**AVM**	**NVM**
Haahr et al.	Nugent score	2	36	12	72
Selim et al.	Nugent score	9	21	26	45
Eckert et al.	Nugent score	3	38	10	81
Gaudoin et al.	Nugent score	10	72	40	206
Boomsma et al.	Nugent score	8	59	17	180
Eldivan et al.	Nugent score	4	12	17	28
Moini et al.	Nugent score	15	123	25	311
Moore et al.	Nugent score	2	25	10	81
Ralph et al.	Nugent score	61	176	190	581
Spandorfer et al.	Nugent score	10	175	14	317
Moragianni et al.	Nugent score	10	22	41	70
**Total**		**134**	**759**	**402**	**1972**

Table legend: AVM=Abnormal vaginal microbiota, NVM=Normal vaginal microbiota

Supplementary Material 8. Forest plot. Live birth rate (Total)

Study or Subgroup	BV Events	BV Total	Normal flora Events	Normal flora Total	Weight	Risk Ratio M-H, Random, 95% CI
Bernabeu 2019	1	2	8	27	2.7%	1.69 [0.38, 7.58]
Haahr 2016	2	18	25	57	3.4%	0.25 [0.07, 0.97]
Liversedge 1999	20	75	50	212	18.4%	1.13 [0.72, 1.77]
Mangot-Bertrand 2012	7	28	67	262	10.8%	0.98 [0.50, 1.92]
Moini 2017	11	25	102	311	17.4%	1.34 [0.84, 2.15]
Moore 2000	2	10	25	81	3.7%	0.65 [0.18, 2.33]
Moragianni 2018	10	41	22	70	11.6%	0.78 [0.41, 1.47]
Spandorfer 2001	8	14	123	317	17.2%	1.47 [0.92, 2.37]
Vergaro 2019	11	35	45	114	14.7%	0.80 [0.46, 1.37]
**Total (95% CI)**		**248**		**1451**	**100.0%**	**1.03 [0.79, 1.33]**
Total events	72		467			

Heterogeneity: Tau² = 0.04; Chi² = 11.19, df = 8 (P = 0.19); I² = 28%
Test for overall effect: Z = 0.20 (P = 0.84)

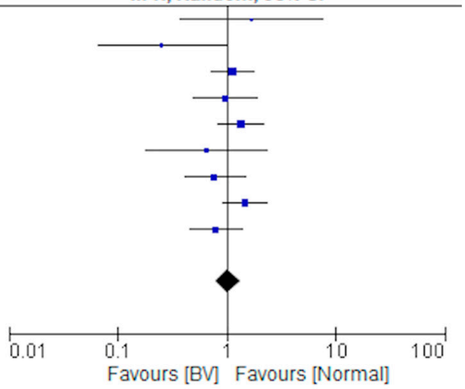

Supplementary Material 8. Forest plot. Live birth rate (Molecular)

Study or Subgroup	BV Events	BV Total	Normal Events	Normal Total	Weight	Risk Ratio M-H, Random, 95% CI
Bernabeu 2019	1	2	8	27	10.5%	1.69 [0.38, 7.58]
Haahr 2016	2	18	25	57	12.8%	0.25 [0.07, 0.97]
Mangot-Bertrand 2012	7	28	67	262	34.0%	0.98 [0.50, 1.92]
Vergaro 2019	11	35	45	114	42.7%	0.80 [0.46, 1.37]
**Total (95% CI)**		**83**		**460**	**100.0%**	**0.80 [0.47, 1.35]**
Total events	21		145			

Heterogeneity: Tau² = 0.09; Chi² = 4.42, df = 3 (P = 0.22); I² = 32%
Test for overall effect: Z = 0.84 (P = 0.40)

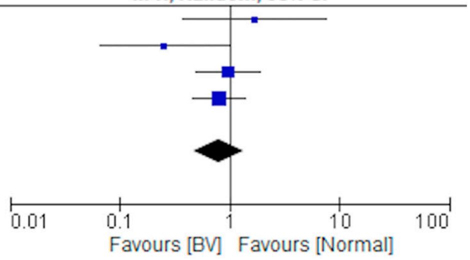

Supplementary Material 8. Forest plot. Live birth rate (Microscopy)

Study or Subgroup	BV Events	BV Total	Normal Events	Normal Total	Weight	Risk Ratio M-H, Random, 95% CI
Haahr 2016	1	11	26	64	2.6%	0.22 [0.03, 1.48]
Liversedge 1999	20	75	50	212	26.0%	1.13 [0.72, 1.77]
Moini 2017	11	25	102	311	24.7%	1.34 [0.84, 2.15]
Moore 2000	2	10	25	81	5.4%	0.65 [0.18, 2.33]
Moragianni 2018	10	41	22	70	16.8%	0.78 [0.41, 1.47]
Spandorfer 2001	8	14	123	317	24.4%	1.47 [0.92, 2.37]
**Total (95% CI)**		**176**		**1055**	**100.0%**	**1.10 [0.80, 1.50]**
Total events	52		348			

Heterogeneity: Tau² = 0.05; Chi² = 7.39, df = 5 (P = 0.19); I² = 32%
Test for overall effect: Z = 0.58 (P = 0.56)

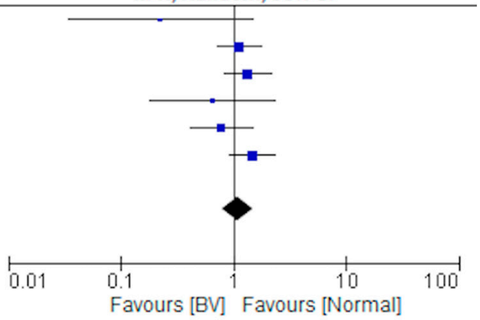

Supplementary Material 9. Forest plot. Early pregnancy loss (Total)

Study or Subgroup	BV Events	BV Total	Normal flora Events	Normal flora Total	Weight	Risk Ratio M-H, Random, 95% CI
Bernabeu 2019	0	2	1	15	0.9%	1.78 [0.09, 34.09]
Boomsma 2010	2	8	18	59	5.0%	0.82 [0.23, 2.89]
Eckert 2003	1	3	13	38	2.9%	0.97 [0.19, 5.12]
Eldivan 2016	0	4	0	12		Not estimable
Gaudoin 1999	3	10	19	72	7.6%	1.14 [0.41, 3.16]
Haahr 2016	4	6	5	32	8.2%	4.27 [1.59, 11.41]
Liversedge 1999	4	24	9	64	6.8%	1.19 [0.40, 3.49]
Mangot-Bertrand 2012	1	8	25	101	2.3%	0.51 [0.08, 3.26]
Moini 2017	4	15	19	123	9.1%	1.73 [0.68, 4.40]
Moragianni 2018	0	41	0	70		Not estimable
Ralph 1999	22	61	34	176	39.1%	1.87 [1.19, 2.93]
Selim 2011	2	9	2	21	2.5%	2.33 [0.39, 14.08]
Spandorfer 2001	2	14	28	317	4.5%	1.62 [0.43, 6.12]
Vergaro 2019	6	17	11	64	11.3%	2.05 [0.89, 4.75]
**Total (95% CI)**		**222**		**1164**	**100.0%**	**1.71 [1.29, 2.27]**
Total events	51		184			

Heterogeneity: Tau² = 0.00; Chi² = 8.45, df = 11 (P = 0.67); I² = 0%
Test for overall effect: Z = 3.73 (P = 0.0002)

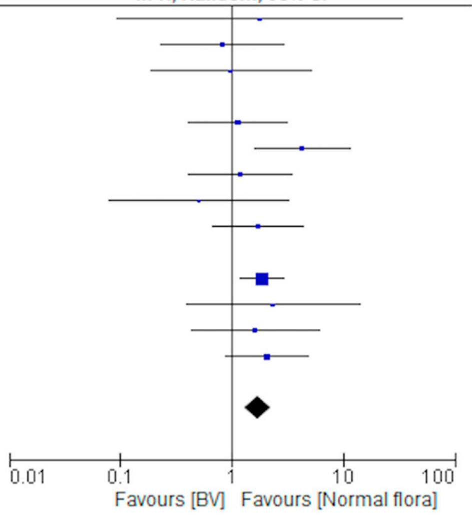

Supplementary Material 9. Forest plot. Early pregnancy loss (Molecular)

Study or Subgroup	BV Events	BV Total	Normal Events	Normal Total	Weight	Risk Ratio M-H, Random, 95% CI
Bernabeu 2019	0	2	1	15	7.2%	1.78 [0.09, 34.09]
Haahr 2016	4	6	5	32	35.7%	4.27 [1.59, 11.41]
Mangot-Bertrand 2012	1	8	25	101	15.7%	0.51 [0.08, 3.26]
Vergaro 2019	6	17	11	64	41.3%	2.05 [0.89, 4.75]
**Total (95% CI)**		**33**		**212**	**100.0%**	**2.12 [0.91, 4.90]**
Total events	11		42			

Heterogeneity: Tau² = 0.26; Chi² = 4.75, df = 3 (P = 0.19); I² = 37%
Test for overall effect: Z = 1.75 (P = 0.08)

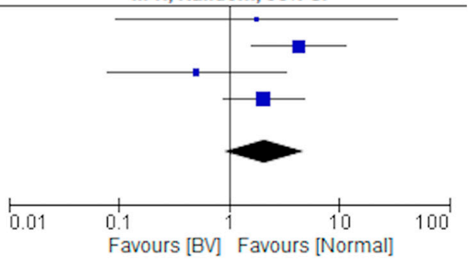

Supplementary Material 9. Forest plot. Early pregnancy loss (Microscopy)

Study or Subgroup	BV Events	BV Total	Normal Events	Normal Total	Weight	Risk Ratio M-H, Random, 95% CI
Boomsma 2010	2	8	18	59	6.2%	0.82 [0.23, 2.89]
Eckert 2003	1	3	13	38	3.6%	0.97 [0.19, 5.12]
Eldivan 2016	0	4	0	12		Not estimable
Gaudoin 1999	3	10	19	72	9.4%	1.14 [0.41, 3.16]
Haahr 2016	1	2	6	36	4.0%	3.00 [0.63, 14.37]
Liversedge 1999	4	24	9	64	8.4%	1.19 [0.40, 3.49]
Moini 2017	4	15	19	123	11.2%	1.73 [0.68, 4.40]
Moragianni 2018	0	41	0	70		Not estimable
Ralph 1999	22	61	34	176	48.5%	1.87 [1.19, 2.93]
Selim 2011	2	9	2	21	3.0%	2.33 [0.39, 14.08]
Spandorfer 2001	2	14	28	317	5.6%	1.62 [0.43, 6.12]
**Total (95% CI)**		**191**		**988**	**100.0%**	**1.61 [1.17, 2.20]**
Total events	41		148			

Heterogeneity: Tau² = 0.00; Chi² = 3.45, df = 8 (P = 0.90); I² = 0%
Test for overall effect: Z = 2.96 (P = 0.003)

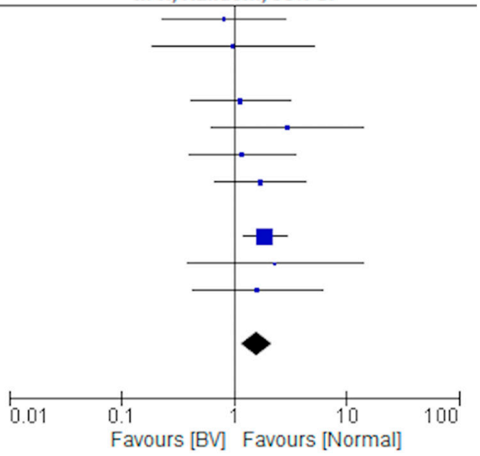

Supplementary Material 10. Forest plot. Clinical pregnancy (Total)

Study or Subgroup	BV Events	BV Total	Normal Events	Normal Total	Weight	Risk Ratio M-H, Random, 95% CI
Bernabeu 2019	1	2	15	29	2.0%	0.97 [0.23, 4.04]
Boomsma 2010	6	17	41	180	6.0%	1.55 [0.77, 3.11]
Eckert 2003	2	10	25	81	2.4%	0.65 [0.18, 2.33]
Eldivan 2016	4	17	12	28	3.8%	0.55 [0.21, 1.43]
Gaudoin 1999	7	40	53	206	5.8%	0.68 [0.33, 1.39]
Haahr 2016	2	22	27	62	2.2%	0.21 [0.05, 0.81]
Koedooder 2019	2	34	65	158	2.1%	0.14 [0.04, 0.56]
Kyono 2018	5	35	9	44	3.6%	0.70 [0.26, 1.90]
Liversedge 1999	24	75	64	212	10.7%	1.06 [0.72, 1.56]
Mangot-Bertrand 2012	8	28	92	262	7.1%	0.81 [0.44, 1.50]
Moini 2017	12	25	108	311	9.8%	1.38 [0.89, 2.14]
Moore 2000	2	10	25	81	2.4%	0.65 [0.18, 2.33]
Moragianni 2018	10	41	22	70	6.7%	0.78 [0.41, 1.47]
Ralph 1999	41	190	122	493	12.3%	0.87 [0.64, 1.19]
Selim 2011	7	26	19	45	5.7%	0.64 [0.31, 1.31]
Spandorfer 2001	8	14	147	317	9.2%	1.23 [0.77, 1.97]
Vergaro 2019	11	35	53	115	8.2%	0.68 [0.40, 1.16]
**Total (95% CI)**		**621**		**2694**	**100.0%**	**0.84 [0.68, 1.04]**
Total events	152		899			

Heterogeneity: Tau² = 0.07; Chi² = 27.24, df = 16 (P = 0.04); I² = 41%
Test for overall effect: Z = 1.63 (P = 0.10)

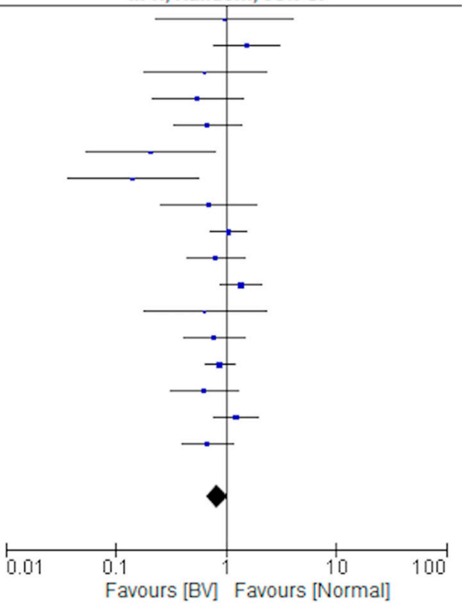

Supplementary Material 10. Forest plot. Clinical pregnancy (Molecular)

Study or Subgroup	BV Events	BV Total	Normal Events	Normal Total	Weight	Risk Ratio M-H, Random, 95% CI
Bernabeu 2019	1	2	15	29	10.1%	0.97 [0.23, 4.04]
Haahr 2016	2	22	27	62	10.9%	0.21 [0.05, 0.81]
Koedooder 2019	2	34	65	158	10.8%	0.14 [0.04, 0.56]
Kyono 2018	5	35	9	44	16.0%	0.70 [0.26, 1.90]
Mangot-Bertrand 2012	8	28	92	262	25.0%	0.81 [0.44, 1.50]
Vergaro 2019	11	35	53	115	27.2%	0.68 [0.40, 1.16]
**Total (95% CI)**		**156**		**670**	**100.0%**	**0.55 [0.32, 0.93]**
Total events	29		261			

Heterogeneity: Tau² = 0.20; Chi² = 9.84, df = 5 (P = 0.08); I² = 49%
Test for overall effect: Z = 2.21 (P = 0.03)

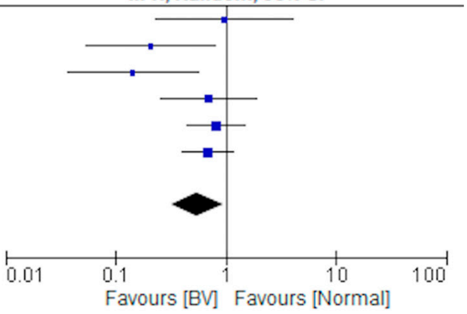

Supplementary Material 10. Forest plot. Clinical pregnancy (Microscopy)

Study or Subgroup	BV Events	BV Total	Normal Events	Normal Total	Weight	Risk Ratio M-H, Random, 95% CI
Boomsma 2010	6	17	41	180	6.8%	1.55 [0.77, 3.11]
Eckert 2003	2	10	25	81	2.3%	0.65 [0.18, 2.33]
Eldivan 2016	4	17	12	28	3.9%	0.55 [0.21, 1.43]
Gaudoin 1999	7	40	53	206	6.5%	0.68 [0.33, 1.39]
Haahr 2016	1	12	28	72	1.1%	0.21 [0.03, 1.43]
Liversedge 1999	24	75	64	212	16.1%	1.06 [0.72, 1.56]
Moini 2017	12	25	108	311	13.9%	1.38 [0.89, 2.14]
Moore 2000	2	10	25	81	2.3%	0.65 [0.18, 2.33]
Moragianni 2018	10	41	22	70	7.8%	0.78 [0.41, 1.47]
Ralph 1999	41	190	122	493	20.5%	0.87 [0.64, 1.19]
Selim 2011	7	26	19	45	6.4%	0.64 [0.31, 1.31]
Spandorfer 2001	8	14	147	317	12.5%	1.23 [0.77, 1.97]
**Total (95% CI)**		**477**		**2096**	**100.0%**	**0.95 [0.78, 1.16]**
Total events	124		666			

Heterogeneity: Tau² = 0.02; Chi² = 14.02, df = 11 (P = 0.23); I² = 22%
Test for overall effect: Z = 0.51 (P = 0.61)

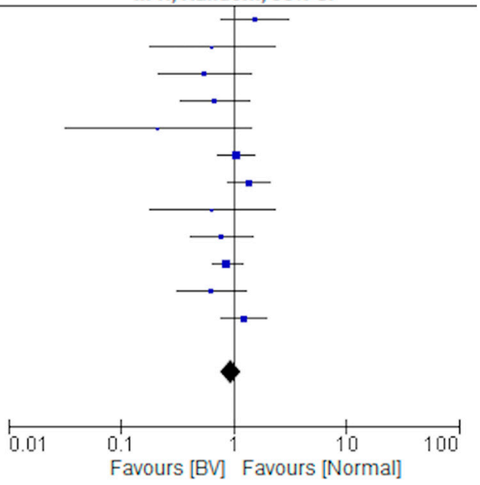

Supplementary Material 11. Forest plot. Biochemical pregnancy (Total)

Study or Subgroup	BV Events	BV Total	Normal microbiota Events	Normal microbiota Total	Weight	Risk Ratio M-H, Random, 95% CI
Bernabeu 2019	1	2	16	29	1.6%	0.91 [0.22, 3.77]
Boomsma 2010	8	17	59	180	7.5%	1.44 [0.83, 2.48]
Eckert 2003	3	10	38	81	3.1%	0.64 [0.24, 1.69]
Eldivan 2016	4	17	12	28	3.2%	0.55 [0.21, 1.43]
Gaudoin 1999	10	40	72	206	7.1%	0.72 [0.41, 1.26]
Haahr 2016	6	22	32	62	5.0%	0.53 [0.26, 1.09]
Mangot-Bertrand 2012	8	28	101	262	6.5%	0.74 [0.40, 1.36]
Moini 2017	15	25	123	311	12.3%	1.52 [1.07, 2.15]
Moore 2000	2	10	25	81	1.9%	0.65 [0.18, 2.33]
Moragianni 2018	10	41	22	70	6.0%	0.78 [0.41, 1.47]
Ralph 1999	61	190	176	581	15.9%	1.06 [0.83, 1.35]
Selim 2011	9	26	21	45	6.4%	0.74 [0.40, 1.37]
Spandorfer 2001	10	14	175	317	12.3%	1.29 [0.92, 1.83]
Vergaro 2019	17	35	64	115	11.4%	0.87 [0.60, 1.27]
**Total (95% CI)**		**477**		**2368**	**100.0%**	**0.95 [0.79, 1.15]**
Total events	164		936			

Heterogeneity: Tau² = 0.04; Chi² = 20.95, df = 13 (P = 0.07); I² = 38%
Test for overall effect: Z = 0.51 (P = 0.61)

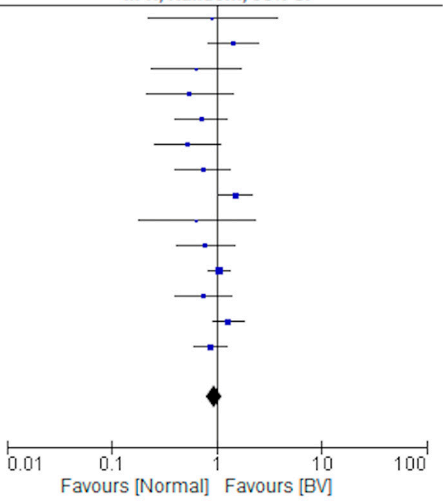

Supplementary Material 11. Forest plot. Biochemical pregnancy (Molecular)

Study or Subgroup	BV Events	BV Total	Normal Events	Normal Total	Weight	Risk Ratio M-H, Random, 95% CI
Bernabeu 2019	1	2	16	29	4.1%	0.91 [0.22, 3.77]
Haahr 2016	6	22	32	62	15.7%	0.53 [0.26, 1.09]
Mangot-Bertrand 2012	8	28	101	262	22.5%	0.74 [0.40, 1.36]
Vergaro 2019	17	35	64	115	57.7%	0.87 [0.60, 1.27]
**Total (95% CI)**		**87**		**468**	**100.0%**	**0.78 [0.58, 1.04]**
Total events	32		213			

Heterogeneity: Tau² = 0.00; Chi² = 1.58, df = 3 (P = 0.66); I² = 0%
Test for overall effect: Z = 1.71 (P = 0.09)

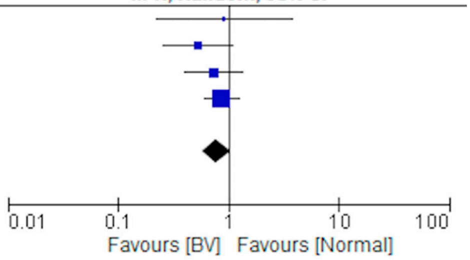

Supplementary Material 11. Forest plot. Biochemical pregnancy (Microscopy)

Study or Subgroup	BV Events	BV Total	Normal Events	Normal Total	Weight	Risk Ratio M-H, Random, 95% CI
Boomsma 2010	8	17	59	180	10.0%	1.44 [0.83, 2.48]
Eckert 2003	3	10	38	81	4.5%	0.64 [0.24, 1.69]
Eldivan 2016	4	17	12	28	4.6%	0.55 [0.21, 1.43]
Gaudoin 1999	10	40	72	206	9.6%	0.72 [0.41, 1.26]
Haahr 2016	2	12	36	72	2.8%	0.33 [0.09, 1.21]
Moini 2017	15	25	123	311	15.1%	1.52 [1.07, 2.15]
Moore 2000	2	10	25	81	2.8%	0.65 [0.18, 2.33]
Moragianni 2018	10	41	22	70	8.3%	0.78 [0.41, 1.47]
Ralph 1999	61	190	176	581	18.4%	1.06 [0.83, 1.35]
Selim 2011	9	26	21	45	8.7%	0.74 [0.40, 1.37]
Spandorfer 2001	10	14	175	317	15.2%	1.29 [0.92, 1.83]
**Total (95% CI)**		**402**		**1972**	**100.0%**	**0.98 [0.78, 1.23]**
Total events	134		759			

Heterogeneity: Tau² = 0.06; Chi² = 18.93, df = 10 (P = 0.04); I² = 47%
Test for overall effect: Z = 0.18 (P = 0.86)

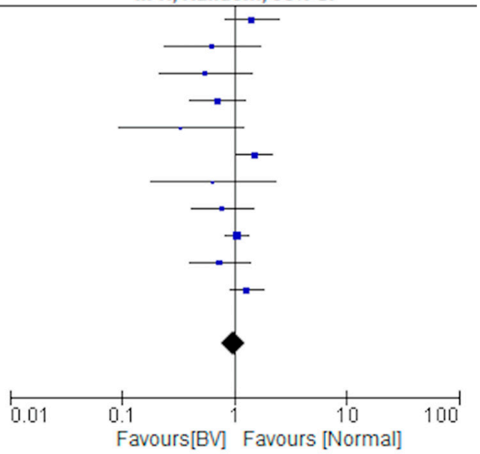

Supplementary Material 12. Funnel plot.

Live birth rate

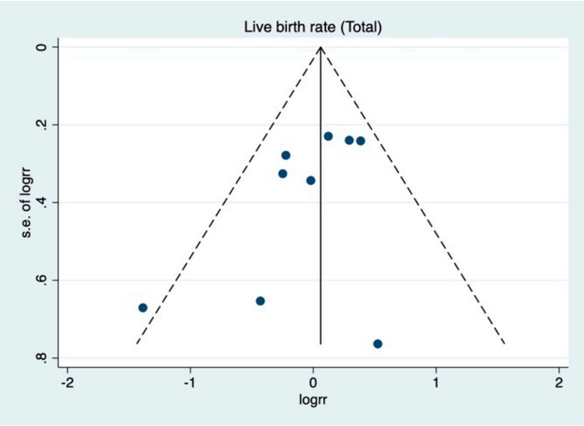

Funnel plot. Live birth rate (Total).
Egger's test: Bias coef. -1.6 (95%CI -3.8-0.6), P=0.135.

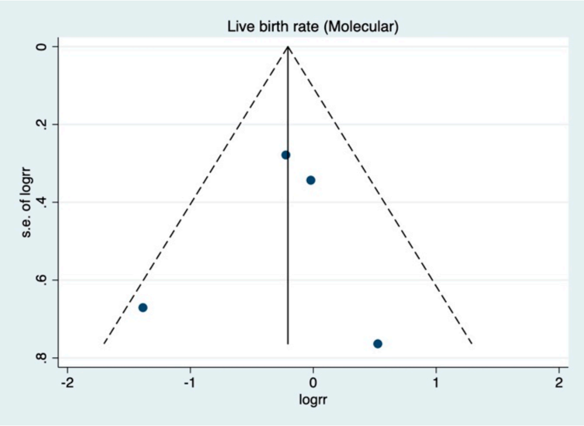

Funnel plot. Live birth rate (Molecular).
Egger's test: Bias coef. -0.5 (95%CI -8.7-7.6), P=0.799

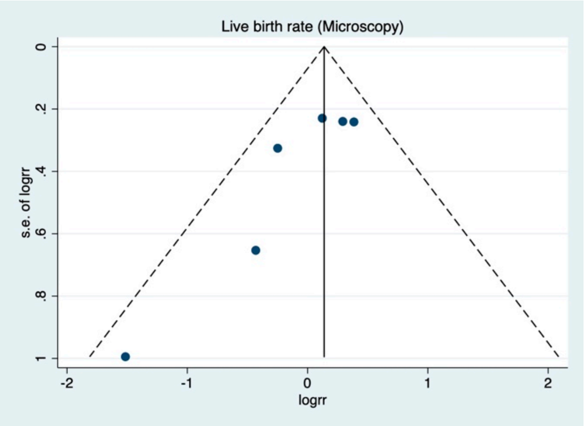

Funnel plot. Live birth rate (Microscopy).
Egger's test: Bias coef. -2.2 (95%CI -4.1- -0.3), P=0.031

Early pregnancy loss.

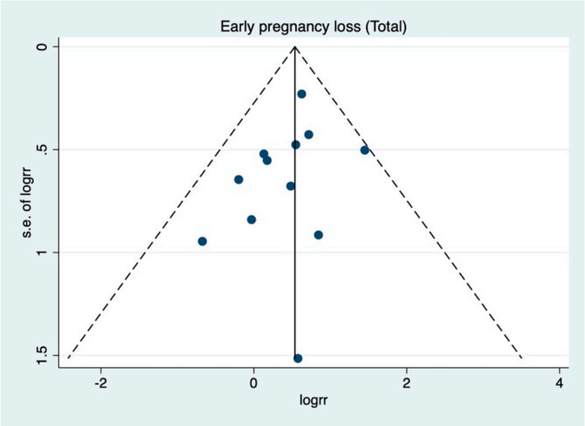

Funnel plot. Early pregnancy loss (Total).
Egger's test: Bias coef. -0.66 (95%CI -1.9-0.6), P=0.254

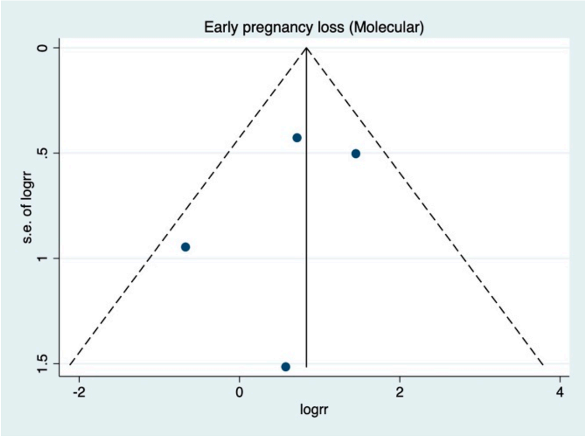

Funnel plot. Early pregnancy loss (Molecular).
Egger's test: Bias coef. -1.2 (95%CI -7.8-5.4), P=0.514

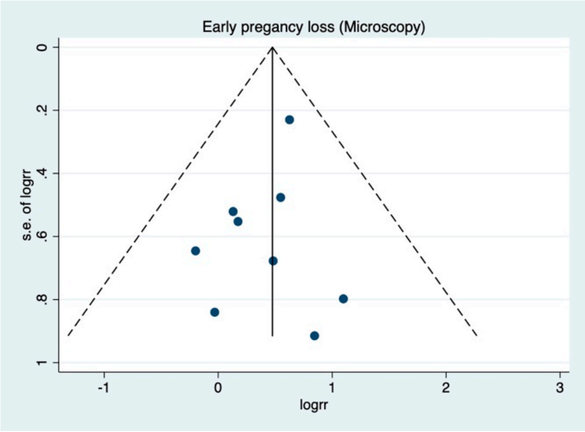

Funnel plot. Early pregnancy loss (Microscopy).
Egger's test: Bias coef. -0.5 (95%CI -1.6-0.7), P=0.366

Clinical pregnancy.

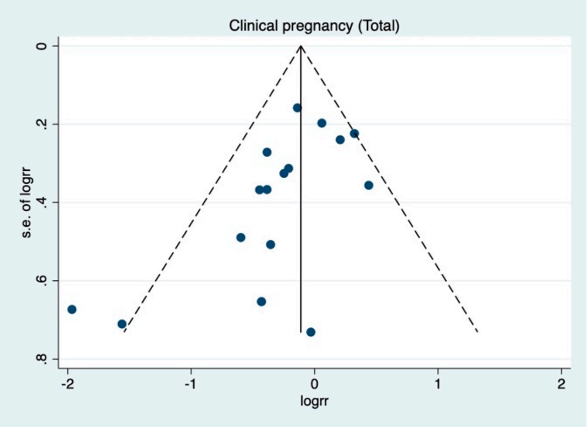

Funnel plot. Clinical pregnancy (Total).
Egger's test: Bias coef. -1.6 (95%CI -2.9- -0.3), P=0.017

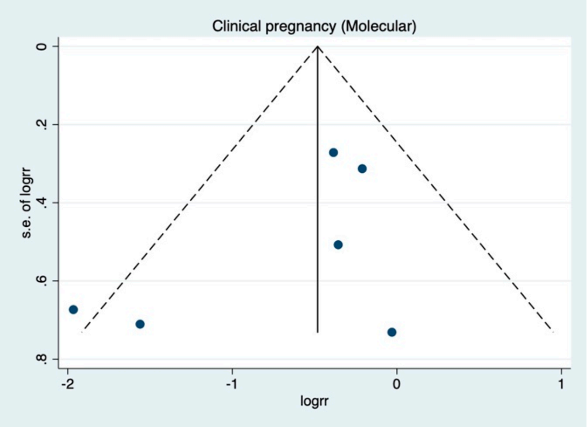

Funnel plot. Clinical pregnancy (Molecular).
Egger's test: Bias coef. -1.8 (95%CI -5,2-1.6), P=0.217

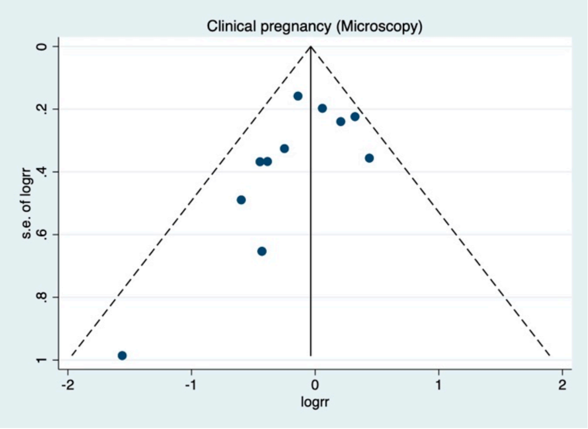

Funnel plot. Clinical pregnancy (Microscopy).
Egger's test: Bias coef. -1.2 (95%CI -2.7-0.2), P=0.083

Biochemical pregnancy.

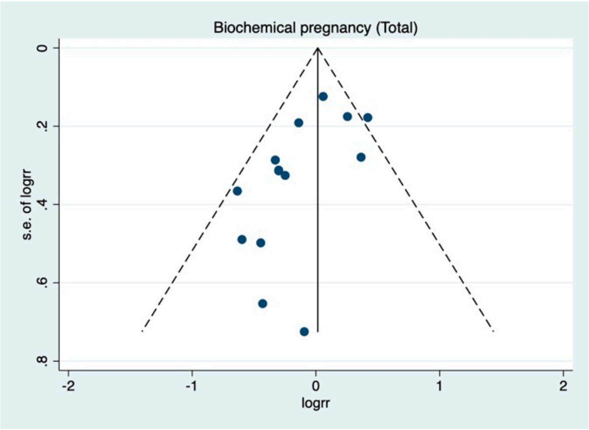

Funnel plot. Biochemical pregnancy (Total).
Egger's test: Bias coef. -1.6 (95%CI -2.9- -0.2), P=0.026

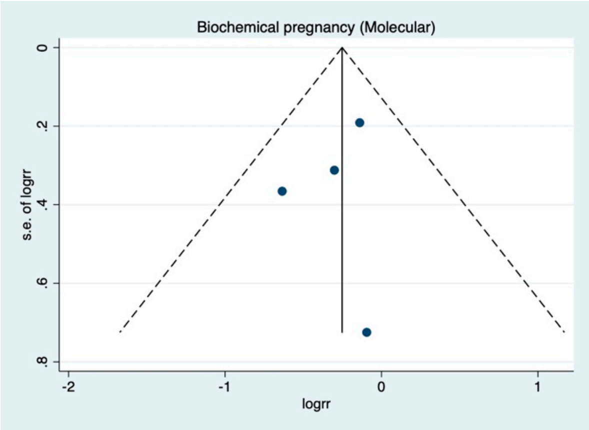

Funnel plot. Biochemical pregnancy (Molecular).
Egger's test: Bias coef. -0.6 (95%CI -4.9-3.6), P=0.596

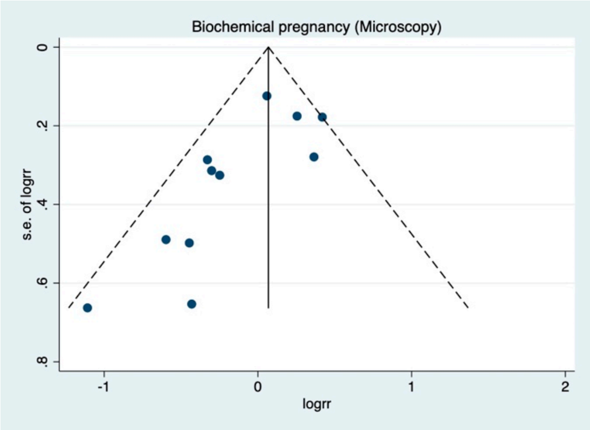

Funnel plot. Clinical pregnancy (Microscopy).
Egger's test: Bias coef. -1.8 (95%CI -3.2- -0.3), P=0.021

Supplementary Material 13. Moose guidelines and PRISMA Guideline.

MOOSE guideline

Reporting Criteria	Reported	Page number
**Reporting of Background**		
Problem definition	Yes	1+2
Hypothesis statement	Yes	1+2
Description of Study Outcome(s)	Yes	10
Type of exposure or intervention used	Yes	10
Type of study design used	Yes	11
Study population	Yes	11
**Reporting of Search Strategy**		
Qualifications of searchers (eg, librarians and investigators)	Yes	11
Search strategy, including time period included in the synthesis and keywords	Yes	11
Effort to include all available studies, including contact with authors	Yes	11
Databases and registries searched	Yes	11
Search software used, name and version, including special features used (eg, explosion)	Yes	12
Use of hand searching (eg, reference lists of obtained articles)	No	
List of citations located and those excluded, including justification	Yes	2+11
Method for addressing articles published in languages other than English	No	
Method of handling abstracts and unpublished studies	Yes	11
Description of any contact with authors	Yes	11
**Reporting of Methods**		
Description of relevance or appropriateness of studies assembled for assessing the hypothesis to be tested	Yes	11
Rationale for the selection and coding of data (eg, sound clinical principles or convenience)	Yes	11
Documentation of how data were classified and coded (eg, multiple raters, blinding, and interrater reliability)	Yes	11
Assessment of confounding (eg, comparability of cases and controls in studies where appropriate	Yes	11
Assessment of study quality, including blinding of quality assessors; stratification or regression on possible predictors of study results	Yes	11
Assessment of heterogeneity	Yes	12
Description of statistical methods (eg, complete description of fixed or random effects models, justification of whether the chosen models account for predictors of study results, dose-response models, or cumulative meta-analysis) in sufficient detail to be replicated	Yes	12
Provision of appropriate tables and graphics	No	
**Reporting of Results**		
Table giving descriptive information for each study included	Yes	5
Results of sensitivity testing (eg, subgroup analysis)	Yes	7 + Supplementary Material
Indication of statistical uncertainty of findings	Yes	4+7
**Reporting of Discussion**		
Quantitative assessment of bias (eg, publication bias)	Yes	8
Justification for exclusion (eg, exclusion of non–English-language citations)	No	

Assessment of quality of included studies	Yes	8
**Reporting of Conclusions**		
Consideration of alternative explanations for observed results	Yes	12
Generalization of the conclusions (ie, appropriate for the data presented and within the domain of the literature review)	Yes	12
Guidelines for future research	Yes	12
Disclosure of funding source	Yes	13

PRISMA Guideline

**Section/Topic**	#	**CHECKLIST ITEM**	**Reported on page #**
		**TITLE**	
Title	1	Identify the report as a systematic review, meta-analysis, or both.	1
		**ABSTRACT**	
Structured summary	2	Provide a structured summary including, as applicable: background; objectives; data sources; study eligibility criteria, participants, and interventions; study appraisal and synthesis methods; results; limitations; conclusions and implications of key findings; systematic review registration number.	1
		**INTRODUCTION**	
Rationale	3	Describe the rationale for the review in the context of what is already known.	2
Objectives	4	Provide an explicit statement of questions being addressed with reference to participants, interventions, comparisons, outcomes, and study design (PICOS).	2
		**METHODS**	
Protocol and registration	5	Indicate if a review protocol exists, if and where it can be accessed (e.g., Web address), and, if available, provide registration information including registration number.	10
Eligibility criteria	6	Specify study characteristics (e.g., PICOS, length of follow-up) and report characteristics (e.g., years considered, language, publication status) used as criteria for eligibility, giving rationale.	11
Information sources	7	Describe all information sources (e.g., databases with dates of coverage, contact with study authors to identify additional studies) in the search and date last searched.	11
Search	8	Present full electronic search strategy for at least one database, including any limits used, such that it could be repeated.	11
Study selection	9	State the process for selecting studies (i.e., screening, eligibility, included in systematic review, and, if applicable, included in the meta-analysis).	11
Data collection process	10	Describe method of data extraction from reports (e.g., piloted forms, independently, in duplicate) and any processes for obtaining and confirming data from investigators.	11
Data items	11	List and define all variables for which data were sought (e.g., PICOS, funding sources) and any assumptions and simplifications made.	11
Risk of bias in individual studies	12	Describe methods used for assessing risk of bias of individual studies (including specification of whether this was done at the study or outcome level), and how this information is to be used in any data synthesis.	11+12

Summary measures	13	State the principal summary measures (e.g., risk ratio, difference in means).	12
Synthesis of results	14	Describe the methods of handling data and combining results of studies, if done, including measures of consistency (e.g., I^2) for each meta-analysis.	12
Risk of bias across studies	15	Specify any assessment of risk of bias that may affect the cumulative evidence (e.g., publication bias, selective reporting within studies).	12
Additional analyses	16	Describe methods of additional analyses (e.g., sensitivity or subgroup analyses, meta-regression), if done, indicating which were pre-specified.	12
**RESULTS**			
Study selection	17	Give numbers of studies screened, assessed for eligibility, and included in the review, with reasons for exclusions at each stage, ideally with a flow diagram.	2
Study characteristics	18	For each study, present characteristics for which data were extracted (e.g., study size, PICOS, follow-up period) and provide the citations.	4
Risk of bias within studies	19	Present data on risk of bias of each study and, if available, any outcome level assessment (see item 12).	4+7+8
Results of individual studies	20	For all outcomes considered (benefits or harms), present, for each study: (a) simple summary data for each intervention group (b) effect estimates and confidence intervals, ideally with a forest plot.	7
Synthesis of results	21	Present results of each meta-analysis done, including confidence intervals and measures of consistency.	4+7+8
Risk of bias across studies	22	Present results of any assessment of risk of bias across studies (see Item 15).	7
Additional analysis	23	Give results of additional analyses, if done (e.g., sensitivity or subgroup analyses, meta-regression [see Item 16]).	4+7+8
**DISCUSSION**			
Summary of evidence	24	Summarize the main findings including the strength of evidence for each main outcome; consider their relevance to key groups (e.g., healthcare providers, users, and policy makers).	8
Limitations	25	Discuss limitations at study and outcome level (e.g., risk of bias), and at review-level (e.g., incomplete retrieval of identified research, reporting bias).	8+9
Conclusions	26	Provide a general interpretation of the results in the context of other evidence, and implications for future research.	9+10
**FUNDING**			
Funding	27	Describe sources of funding for the systematic review and other support (e.g., supply of data); role of funders for the systematic review.	13

Supplementary Material 14. GRADE. Quality of evidence.

Outcome	No of studies	Design	Risk of Bias	Inconsistency	Indirectness	Imprecision	Risk of publication bias
**Live birth rate (Total)**	9	Observation studies	High	I^2=28%. The inconsistency was low.	Although all patients were treated with IVF, the indirectness was affected as the populations were different in many ways including origin of country and IVF cycle of sampling.	Imprecision moderate due to a confidence interval that included potential for important harm or benefit.	Risk of publication bias was low according to Egger's test (P=0.135). See Supplementary Material 12.
**Live birth rate (Molecular)**	4	Observation studies	High	I^2=32%. The inconsistency was low.	Although all patients were treated with IVF, the indirectness was affected as the populations were different in many ways including origin of country and IVF cycle of sampling.	Imprecision high due to a confidence interval that included potential for important harm or benefit. Also, small sample size.	As only four studies are included in the Funnel Plot, the power of tests is too low to evaluate the risk of publication bias. See Supplementary Material 12.
**Live birth rate (Microscopy)**	Quality of evidence: Very low. Assessment can be found in our previous study see the Material and Methods section in the Article. The addition of Moragianni et al. did no change the level of evidence.						
**Early pregnancy loss (Total)**	14	Observation studies	High	I^2=0%. The inconsistency was low.	Although all patients were treated with IVF, the indirectness was affected as the populations were different in many ways including origin of country and IVF cycle of sampling.	Imprecision low.	Risk of publication bias was low according to Egger's test (P=0.254) See Supplementary Material 12.
**Early pregnancy loss (Molecular)**	4	Observation studies	High	I^2=37%. The inconsistency was low.	Although all patients were treated with IVF, the indirectness was affected as the populations were different in many ways including origin of country and IVF cycle of sampling.	Imprecision high due to a wide CI interval that included potential for important harm or benefit. Also, small sample size.	As only four studies are included in the Funnel Plot, the power of tests is too low to evaluate the risk of publication bias. See Supplementary Material 12.
**Early pregnancy loss (Microscopy)**	Quality of evidence: Very low. Assessment can be found in our previous study see the Material and Methods section in the Article. The addition of Moragianni et al. did no change the level of evidence.						
**Clinical pregnancy (Total)**	17	Observation studies	High	I^2=41%. The inconsistency was moderate.	Although all patients were treated with IVF, the indirectness was affected as the populations were different in many ways including origin of country and IVF cycle of sampling.	Imprecision moderate due to a confidence interval that included potential for important harm or benefit.	Risk of publication bias high according to Egger's test (P=0.017). See Supplementary Material 12.
**Clinical Pregnancy (Molecular)**	6	Observation studies	High	I^2=49%. The inconsistency was moderate.	Although all patients were treated with IVF, the indirectness was affected as the populations were different in many ways including origin of country and IVF cycle of sampling.	Imprecision low	As only six studies are included in the Funnel Plot, the power of tests is too low to evaluate the risk of publication bias. See Supplementary Material 12.
**Clinical Pregnancy (Microscopy)**	Quality of evidence: Very low. Assessment can be found in our previous study see the Material and Methods section in the Article. The addition of Moragianni et al. did no change the level of evidence.						
**Biochemical pregnancy (Total)**	14	Observation studies	High	I^2=38%. The inconsistency was low.	Although all patients were treated with IVF, the indirectness was affected as the populations were different in many ways including origin of country and IVF cycle of sampling.	Imprecision moderate due to a confidence interval that included potential for important harm or benefit.	Risk of publication bias high according to Egger's test (P=0.026). See Supplementary Material 12.
**Biochemical pregnancy (Molecular)**	4	Observation studies	High	I^2=0%. The inconsist	Although all patients were treated with IVF, the indirectness was affected as the populations were different	Imprecision high due to a wide CI interval that included potential	As only four studies are included in the Funnel Plot, the power of tests is too low to evaluate the risk of

				ency was low.	in many ways including origin of country and IVF cycle of sampling.	for important harm or benefit. Also, small sample size.	publication bias. See Supplementary Material 12.
**Biochemical pregnancy (Microscopy)**	Quality of evidence: Very low. Assessment can be found in our previous study see the Material and Methods section in the Article. The addition of Moragianni et al. did no change the level of evidence.						
